# O-19 - IMPROVING GASTROINTESTINAL INFECTION SURVEILLANCE THROUGH IMPLEMENTATION OF SELF-COMPLETED ONLINE EXPOSURE QUESTIONNAIRES

**DOI:** 10.1093/pubmed/fdag046.016

**Published:** 2026-07-09

**Authors:** Mr Reece Jarratt, Emma Wilson, Sam Rowell, Mark McGivern, Elizabeth Stratford, Pam Griffiths, Katherine Platt, Roberto Vivancos, Emma Savage

**Affiliations:** UK Health Security Agency; NIHR Health Protection research Unit in Gastrointestinal infections, University of East Anglia; UK Health Security Agency; UK Health Security Agency; UK Health Security Agency; UK Health Security Agency; UK Health Security Agency; UK Health Security Agency; UK Health Security Agency; NIHR Health Protection research Unit in Gastrointestinal infections, University of East Anglia; Warwick Medical School, The University of Warwick; UK Health Security Agency

## Abstract

**Background:**

Surveillance of gastrointestinal (GI) infections in England relies on timely and high-quality exposure data to detect outbreaks and emerging risks^1^. Traditionally, Environmental Health Teams (EHTs) aid the completion of exposure questionnaires for low-risk GI cases on behalf of the UK Health Security Agency (UKHSA). This approach was resource intensive, resulted in variable timeliness, and delayed the collection of exposure data necessary for surveillance. A regional consultation across EHTs in the North West identified variation in questionnaire content, delivery methods, and the use of outdated tools.

**Objectives:**

To improve the timeliness, completion, and standardisation of exposure data collection for low-risk GI cases, while increasing UKHSAs surveillance capacity and reducing operational burden for EHTs.

**Methods:**

A standardised, self-completed online exposure questionnaire was developed for low-risk GI cases. Cases were invited to complete the questionnaire directly, via SMS. The questionnaire was implemented regionally to ensure a consistent approach to data collection. In parallel, a new routine epidemiological output was developed to provide EHTs with regular summaries of low-risk GI activity and reported exposures within their local authority.

**Results:**

Early findings indicate an improvement in response rates between 10 to 22% compared with the one year prior. Timeliness of data collection improved substantially, with the average time from case notification to questionnaire completion reduced by over half compared to 2023, when the mean interval was approximately seven days. The transition to standardised electronic data capture reduced information governance risks associated with routine case-level data sharing. Newly developed epidemiological outputs enhanced local situational awareness and were positively received.

**Conclusions:**

The introduction of self-completed online questionnaires for low-risk GI cases has strengthened surveillance through improved timeliness, standardisation, and data governance. This approach supports more efficient regional surveillance while maintaining effective collaboration partners. Further evaluation will assess data quality, completeness, and timeliness to inform national implementation.

References

1. UK Health Security Agency. *Gastrointestinal infections in England*. London: UKHSA; [Accessed 08 April 2026]. Available from: https://www.gov.uk/government/publications/gastrointestinal-infections-in-england

